# Nutrition Training Improves Health Workers’ Nutrition Knowledge and Competence to Manage Child Undernutrition: A Systematic Review

**DOI:** 10.3389/fpubh.2013.00037

**Published:** 2013-09-24

**Authors:** Bruno F. Sunguya, Krishna C. Poudel, Linda B. Mlunde, David P. Urassa, Junko Yasuoka, Masamine Jimba

**Affiliations:** ^1^Department of Community and Global Health, Graduate School of Medicine, The University of Tokyo, Tokyo, Japan; ^2^Department of Public Health, School of Public Health and Health Sciences, University of Massachusetts Amherst, Amherst, MA, USA; ^3^School of Public Health and Social Sciences, Muhimbili University of Health and Allied Sciences, Dar es Salaam, Tanzania

**Keywords:** in-service training, nutritional sciences, health knowledge, counseling, child undernutrition, health personnel

## Abstract

**Background:** Medical and nursing education lack adequate practical nutrition training to fit the clinical reality that health workers face in their practices. Such a deficit creates health workers with poor nutrition knowledge and child undernutrition management practices. In-service nutrition training can help to fill this gap. However, no systematic review has examined its collective effectiveness. We thus conducted this study to examine the effectiveness of in-service nutrition training on health workers’ nutrition knowledge, counseling skills, and child undernutrition management practices.

**Methods:** We conducted a literature search on nutrition interventions from PubMed/MEDLINE, CINAHL, EMBASE, ISI Web of Knowledge, and World Health Organization regional databases. The outcome variables were nutrition knowledge, nutrition-counseling skills, and undernutrition management practices of health workers. Due to heterogeneity, we conducted only descriptive analyses.

**Results:** Out of 3910 retrieved articles, 25 were selected as eligible for the final analysis. A total of 18 studies evaluated health workers’ nutrition knowledge and showed improvement after training. A total of 12 studies with nutrition counseling as the outcome variable also showed improvement among the trained health workers. Sixteen studies evaluated health workers’ child undernutrition management practices. In all such studies, child undernutrition management practices and competence of health workers improved after the nutrition training intervention.

**Conclusion:** In-service nutrition training improves quality of health workers by rendering them more knowledge and competence to manage nutrition-related conditions, especially child undernutrition. In-service nutrition training interventions can help to fill the gap created by the lack of adequate nutrition training in the existing medical and nursing education system. In this way, steps can be taken toward improving the overall nutritional status of the child population.

## Background

Child undernutrition can be reduced if health workers with adequate nutrition knowledge provide correct, adequate, and frequent nutrition advise to caregivers (1, 2). Across the globe, the quality of health workers’ nutrition knowledge – and, by extension, their counseling skills – has been a concern (3–7). Historically, medical training has lacked adequate and updated nutrition training that is in keeping with the situation and needs on the ground (8–10). As a result, health workers produced from teaching institutions have lacked adequate nutrition knowledge (3, 6). Such health workers may also lack the competence and skills to provide basic nutrition advice to their clients (2, 11). This incompetence, in turn, may be a factor deterring health workers from providing nutrition advice and management to their clients (12).

In-service nutrition training can help to improve health workers’ nutrition knowledge (13–16). This may facilitate positive changes in their attitudes toward nutrition care (17, 18) and thus in their behavior (19–21). As a result, health workers’ skills in management of nutrition-related problems such as child undernutrition, including nutrition-counseling skills (13–15, 19, 22, 23), may improve (24–26).

In practical terms, the process by which the knowledge acquired through nutrition training is translated into management practices may not be linear. However, the outcome of nutrition training can be explained using the conceptual framework of general behavioral theories. Based on the Health Belief Model (27), for example, knowledge or education on a perceived threat or disease is likely to influence behavior change. Untrained health workers may feel incompetent to provide counseling to their clients even when they know or perceive the threat caused by a nutrition problem. When such health workers are trained to recognize threats posed by nutrition-related problems, they are likely to provide appropriate care. In an ideal situation, knowledge can impact practical skills; it can thus change health workers’ behaviors.

Changes in health workers’ nutrition-counseling behavior can be sustained even after training ends. This may be due to the rewards they may receive in the positive results of improving child feeding practices and nutritional status. According to Bandura’s social learning theory, a change can be influenced by being rewarded or punished as a result of one’s actions (27). Health workers’ counseling behavior is more likely to last or recur if the nutrition knowledge received is used to counsel or manage a child with undernutrition so as to yield obvious improvements in the child’s nutritional status.

Evidence is available for the impact of in-service nutrition training on health workers’ nutrition knowledge, nutrition-counseling skills, and management of child undernutrition. However, no systematic review has summarized such evidence toward effecting policy change. A few review articles have demonstrated the importance of nutrition training for health workers (7). Other reviews have shown the effect of health workers’ counseling of caregivers on feeding practices including dietary diversity, feeding frequency, and energy intake (28), complementary feeding, and children’s nutritional status (1, 29, 30). However, we could not find any systematic review on the impact of nutrition training for health workers on their own knowledge, attitudes, and child undernutrition management practices. Therefore the objective of this systematic review was to evaluate the impact of nutrition training interventions among health workers on their nutrition knowledge, nutrition counseling, and child undernutrition management practices.

## Methods

The population, intervention, comparator, outcome (PICO) question to be addressed in this study was framed as follows: what is the effectiveness of nutrition training of health workers on their nutrition knowledge, nutrition counseling, and undernutrition management practices among children at risk of or suffering from undernutrition as compared to those who did not receive such training? For this review, we included studies with nutrition training interventions.

We defined nutrition training for health workers as any form of in-service training given to health workers and designed for continuing professional development (CPD), continuous medical education (CME), research purposes, or as part of a health project or program. We used a World Health Organization (WHO) and International Labor Organization (ILO) definitions of health worker to select health cadres as the population of interest (31, 32). Such health cadres included doctors, nurses, midwives, mid-level providers, dieticians, nutritionists, and pharmacists.

Three outcome variables were assessed in this review: health worker’s nutrition knowledge, nutrition counseling and/or general counseling skills, and management skills for child undernutrition. Health worker’s nutrition knowledge was measured using a standard scale or pre-made set of questions to test knowledge specific to the training given. We regarded nutrition counseling as any specific advice given by a health worker to caregivers on nutrition, feeding characteristics, dietary composition, or food intake. Such advice might have followed evaluation of the patient’s nutritional status, feeding behavior, dietary composition, or training on poor nutrition conditions. Depending on the availability of the data, we included assessment of quality and frequency of counseling to assess both skills and counseling acts of trained health workers.

We defined management practices for undernutrition as activities health workers perform toward management of poor nutritional status. Depending on data availability, this might include assessment of undernutrition using anthropometric scores, assessment of micronutrient deficiency, treatment of associated conditions, treatment of undernutrition by prescription of supplements, or monitoring by growth charts, among other methods.

The protocol for this systematic review was registered at PROSPERO http://www.crd.york.ac.uk/PROSPERO on February 6, 2013. The registration number for this review is CDR42013003800. It is available at http://www.crd.york.ac.uk/PROSPERO/display_record.asp?ID=CRD42013003800.

## Inclusion Criteria

Based on the nature of the intervention and the outcome of interest, we included studies with nutrition training interventions. As for the study design, we included studies conducted as RCTs, cluster RCTs, quasi-experimental studies, and pre–post-intervention longitudinal studies with or without comparison groups.

## Exclusion Criteria

We excluded any study where in the structure and/or quality of the training intervention provided to health workers was unclear from the description provided. We also excluded studies involving cadres of health workers outside the scope of the WHO that included community health volunteers, medical and nursing students, medical interns, and peers trained to provide specified health services. Non-interventional studies were also excluded from this review. This included cross-sectional studies, case reports, and non-interventional qualitative studies.

## Data Sources for Existing Reviews

We first searched for similar reviews or registered review protocols to avoid duplication and redundancy according to the standard review guidelines of the Center for Review and Dissemination (CRD) and Cochrane (33, 34). To this end, we searched such protocols listed in the Cochrane library and Cochrane Database of Systematic Reviews (CDSR). We used a similar approach to search other important databases for systematic reviews, including the Database of Abstracts of Reviews of Effects (DARE), the Educational Resources and Information Center (ERIC), the Campbell Library of Systematic Reviews, and the National Institute for Health and Clinical Excellence (NICE). Two independent researchers conducted the search for existing protocols and similar review articles.

## Evidence Search Strategy

**Figure 1:**
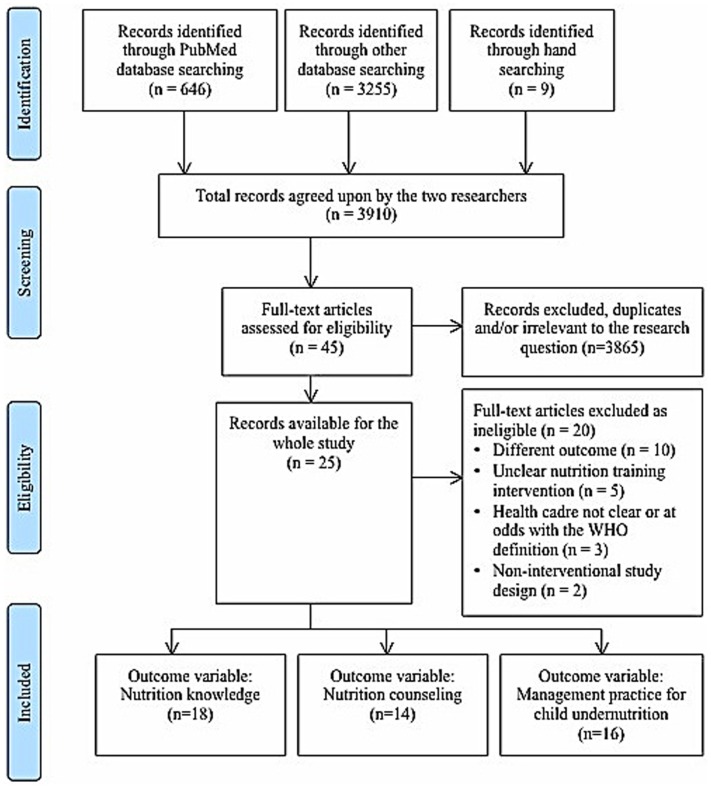
**Diagram of information flow through phases of systematic review**.

Two independent researchers conducted a literature search based on the published review protocol. The search was conducted in five medical databases: PubMed/Medline, CINAHL, EMBASE, ISI Web of Knowledge, and WHO regional databases. The search was limited to a 15-year-publication period (1997–2012), to ensure that we obtain enough evidence. Also, most of standard nutrition training interventions became more common after the publication of Integrated Management of Childhood Illnesses (IMCI) by WHO in 1997. For the PubMed/Medline database, we followed the search strategy outlined in the CDR register. Similar key words were then used to conduct searches in other selected databases. In order to ensure that we captured most of the relevant articles, we also conducted a hand search using references from key identified articles and archives of a journal with similar specialty – the Journal of Human Resources for Health. Figure 1 shows the results of searching and data management procedures according to the PRISMA check list (Table 1) (35).

**Table 1:** **PRISMA 2009 checklist**.

Section/topic	No.	Checklist item	Reported on page No.
**TITLE**			
Title	1	Identify the report as a systematic review, meta-analysis, or both	1
**ABSTRACT**			
Structured summary	2	Provide a structured summary including, as applicable: background; objectives; data sources; study eligibility criteria, participants, and interventions; study appraisal and synthesis methods; results; limitations; conclusions and implications of key findings; systematic review registration number	2
**INTRODUCTION**			
Rationale	3	Describe the rationale for the review in the context of what is already known	3
Objectives	4	Provide an explicit statement of questions being addressed with reference to participants, interventions, comparisons, outcomes, and study design (PICOS)	4
**METHODS**			
Protocol and registration	5	Indicate if a review protocol exists, if and where it can be accessed (e.g., Web address), and, if available, provide registration information including registration number	4
Eligibility criteria	6	Specify study characteristics (e.g., PICOS, length of follow-up) and report characteristics (e.g., years considered, language, publication status) used as criteria for eligibility, giving rationale	4–5
Information sources	7	Describe all information sources (e.g., databases with dates of coverage, contact with study authors to identify additional studies) in the search and date last searched	5–6
Search	8	Present full electronic search strategy for at least one database, including any limits used, such that it could be repeated	6
Study selection	9	State the process for selecting studies (i.e., screening, eligibility, included in systematic review, and, if applicable, included in the meta-analysis)	4–6
Data collection process	10	Describe method of data extraction from reports (e.g., piloted forms, independently, in duplicate) and any processes for obtaining and confirming data from investigators	5
Data items	11	List and define all variables for which data were sought (e.g., PICOS, funding sources) and any assumptions and simplifications made	5–6
Risk of bias in individual studies	12	Describe methods used for assessing risk of bias of individual studies (including specification of whether this was done at the study or outcome level), and how this information is to be used in any data synthesis	6, Tables 2–3
Summary measures	13	State the principal summary measures (e.g., risk ratio, difference in means)	5–6
Synthesis of results	14	Describe the methods of handling data and combining results of studies, if done, including measures of consistency (e.g., I^2^) for each meta-analysis	5–6
Risk of bias across studies	15	Specify any assessment of risk of bias that may affect the cumulative evidence (e.g., publication bias, selective reporting within studies)	6
Additional analyses	16	Describe methods of additional analyses (e.g., sensitivity or subgroup analyses, meta-regression), if done, indicating which were pre-specified	NA
**RESULTS**			
Study selection	17	Give numbers of studies screened, assessed for eligibility, and included in the review, with reasons for exclusions at each stage, ideally with a flow diagram	6, Figure 1
Study characteristics	18	For each study, present characteristics for which data were extracted (e.g., study size, PICOS, follow-up period) and provide the citations	Table 4
Risk of bias within studies	19	Present data on risk of bias of each study and, if available, any outcome level assessment (see item 12)	Tables 2–3
Results of individual studies	20	For all outcomes considered (benefits or harms), present, for each study: (a) simple summary data for each intervention group (b) effect estimates and confidence intervals, ideally with a forest plot	Tables 5–7
Synthesis of results	21	Present results of each meta-analysis done, including confidence intervals and measures of consistency	NA
Risk of bias across studies	22	Present results of any assessment of risk of bias across studies (see item 15)	Tables 2–3
Additional analysis	23	Give results of additional analyses, if done (e.g., sensitivity or subgroup analyses, meta-regression [see item 16])	NA
**DISCUSSION**			
Summary of evidence	24	Summarize the main findings including the strength of evidence for each main outcome; consider their relevance to key groups (e.g., healthcare providers, users, and policy makers)	8–10
Limitations	25	Discuss limitations at study and outcome level (e.g., risk of bias), and at review-level (e.g., incomplete retrieval of identified research, reporting bias)	9–10
Conclusions	26	Provide a general interpretation of the results in the context of other evidence, and implications for future research	10
**FUNDING**			
Funding	27	Describe sources of funding for the systematic review and other support (e.g., supply of data); role of funders for the systematic review	10

From Ref. (36).

A total of 3910 articles were retrieved from various sources. Of these, 646 studies were from PubMed/Medline. We retrieved a total of 3255 from other databases: 341 from CINAHL, 1543 from EMBASE, 1249 from ISI Web of Knowledge, and 122 from WHO Regional databases. A total of nine studies were additionally obtained based on hand searches and review of specific journal databases.

On an initial screening of articles, we excluded a total of 3865 abstracts and articles due to duplication between PubMed/Medline and other databases and those, which were irrelevant to the research question. Of the 45 studies remaining, we further excluded 20 studies following a full-text assessment of eligibility. The reasons for exclusion were as follows: different outcome variable to that of interest (*n* = 10), unclear or different intervention (*n* = 5), unclear or different population (*n* = 3), and different study design (*n* = 2).

## Analysis Strategy

Also shown in Figure 1 is the distribution of the 25 studies included for final analysis according to outcome variables. A single study could incorporate more than one outcome variable of interest. A total of 18 studies had health workers’ nutrition knowledge as an outcome variable, 12 studies had health worker’s nutrition counseling or general counseling skills as an outcome variable, and 16 studies had health worker’s management practice as an outcome variable.

Of the 25 identified studies, six used a cluster RCT design (13–15, 19, 22, 23). One study had a controlled non-randomized trial design (16). A total of 18 studies, meanwhile, used a pre–post-intervention evaluation and quasi-experimental design with or without a control group (17, 20, 21, 24, 37–49).

The duration of the intervention, population characteristics, and measurement of outcome variables differed across each of the included studies. Study design also differed from one study to another though they all aimed generally at a nutrition training intervention for health workers (Table 4). To avoid the obvious risk of heterogeneity, we did not conduct a meta-analysis. Instead, we sought to write a narrative summary to describe our results and stratified such description according to the outcome of interest.

## Risk of Bias

We assessed risk of bias (RoB) for each cluster RCT and non-randomized studies (Tables 2 and 3). We used the Cochrane RoB tool (34), to examine five types of RoB for randomized trials. These included selection, performance, attrition, detection, and reporting biases. Of the six cluster RCTs, only one study (13), had a high risk of selection and performance bias.

**Table 2:** **Risk of bias assessment cluster RCTs Cluster RCTs**.

	Selection bias: allocation concealment	Performance bias: blinding of participants and personnel	Detection bias: blinding of outcome assessment	Attrition bias: incomplete outcome data	Reporting bias: selective reporting
Zaman et al. (22)					
Bassichetto and Réa (13)	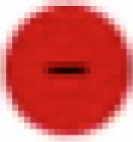	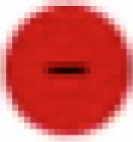			
Moore et al. (14)					
Pelto et al. (19)				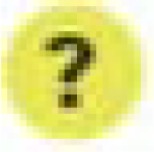	
Santos et al. (15)					
Penny et al. (23)		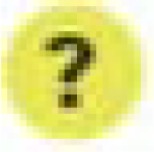			

+ Indicates low risk of bias, − indicates high risk of bias, and ? indicate unclear risk of bias.

**Table 3:** **Risk of Bias (ROB) and methodological quality for non-randomized studies**.

Author	Reporting (10)	External validity (3)	Internal validity-bias (7)	Internal validity-(confounding) selection bias (6)	Power (1)
Palermo et al. (37)	8	3	6	4	No
Lindorff-Larsen et al. (17)	8	3	6	4	No
Puoane et al. (38)	5	2	4	2	No
Hamer et al. (39)	8	1	6	3	Yes
Edwards and Wyles (40)	6	2	5	1	Yes
O’Mahony et al. (41)	8	2	6	4	No
Hillenbrand and Larsen (20)	9	2	6	4	No
Olsson et al. (42)	8	3	6	4	Yes
Pedersen et al. (43)	9	2	6	4	No
Gance-Cleverland et al. (21)	8	3	4	4	No
Bjerrum et al. (18)	5	2	4	2	No
Kennelly et al. (45)	9	3	6	4	Yes
Kennelly et al. (44)	9	3	6	4	Yes
Simoes et al. (46)	9	2	5	4	No
Davies-Adetugbo and Adebawa (47)	9	3	6	4	Yes
Newes-Adeyi et al. (48)	8	2	6	4	No
Stark et al. (49)	9	3	7	2	Yes
Charlton et al. (24)	9	3	6	4	Yes

To assess quality and RoB for non-randomized studies, we used the Downs and Black scoring system (50). This tool has 27 checklists and is used to examine RoB for observation studies. We used the tool to examine reporting bias, external validity, internal validity-bias, internal validity- (confounding) selection bias, and power of the study. Generally, the included non-randomized studies had good external validity and internal validity. However, 10 out of 18 of non-randomized studies included had low or unknown power to detect a clinical important effect of the intervention. This is due to the nature of the intervention and methods used to recruit their participants. Most of nutrition training used all available health workers, selected non-randomly, and without prior or unspecified sample size calculations.

## Results

### General description of the included studies

Out of 3910 retrieved studies, a total of 25 studies were included in this systematic review. These studies were conducted in countries from Africa, North and South America, Europe, Asia, and Australia (Table 4). The selected studies were also conducted in varied socio-economic levels, from least to most developed countries. Among the included studies, six were cluster RCTs, one was a controlled non-randomized trial, 17 were pre–post-intervention evaluations, and one was a quasi-experimental design study. The included health worker cadres ranged from specialists in pediatric care to general practitioners (GPs), nurses, midwives and obstetricians, nutritionists, dieticians, and mid-level providers.

**Table 4:** **General description of studies included in the review**.

Author	Study design	Nutrition training intervention	Outcome of interest
Zaman et al. (22), Pakistan	Cluster RCT	Nutrition-counseling training using IMCI’s “counsel the mother” module for five and a half days. It included infant feeding knowledge and practice sessions for the development of communication and counseling skills	Communication skills
			Nutrition counseling
			Nutrition management/practice
Bassichetto and Réa (13), Brazil	Cluster RCT	WHO’s “infant and young child feeding counseling: an integrated course.” The training includes 8 h of practical sessions. Out of 34 sessions, 8 were dedicated to breastfeeding, 6 to HIV and infant and young child feeding, 7 to complementary feeding, 10 to counseling, and 4 to general themes, making a total of 40 h	Nutrition knowledge
			Nutrition counseling
			Undernutrition management/performance
Moore et al. (14), UK	Cluster RCT	A nutrition training program was delivered to six intervention practices (health facilities). Emphasis of the training was on increasing motivation to improve quality of dietary consultations and providing them with practical skills adapted from behavior models. Included components in the 7.5-h training were patients’ assessment, education, and goal setting in issues of public health importance including drinking	Nutrition knowledge Counseling
Pelto et al. (19), Brazil	Cluster RCT	Physicians from the intervention group received a 20-h training in a program derived from the IMCI nutrition-counseling module. After training, they provided care to caregiver/child pairs who attended their centers	Counseling
			Practice: communication skills
Santos et al. (15), Brazil	Cluster RCT	Fourteen doctors received a 20-h nutrition-counseling training using IMCI’s “counsel the mother” and “management of the sick young infant” modules. Based on local adaptation of IMCI feeding guidelines, the key feeding recommendations identified were as follows: increase breast and complementary feeding frequency, provide animal protein and micronutrient-rich foods, add oil to the food, and increase dietary diversity. Of the 20 h of training, 40% was used for practical sessions in a health center	Nutrition knowledge Nutrition counseling: undernutrition management skills/practice
Penny et al. (23), Peru	Cluster RCT	Interventions aimed to raise the nutrition profile of the health facility and to integrate nutrition services into existing child health programs though training and provision of simple messages to caregivers. Training included demonstration of preparation of complementary foods and child’s age-specific group sessions for their caregivers. The intervention also included training for health care workers to improve anthropometry skills. An accreditation system was also introduced for institutional change	Nutrition counseling Others: health seeking behavior
Cattaneo and Buzzetti (16), Italy	Controlled non-randomized trial	An 18-h UNICEF “Breastfeeding, management, and health proportion in baby-friendly hospitals” course along with a 2-h counseling session from the WHO’s breast-feeding counseling course were implemented	Nutrition knowledge Hospital performance
Palermo et al. (37), Australia	Pre–post-intervention study	Thirty-two dieticians were allocated to three intervention groups: two face-to-face groups and one rural video-linked group. The intervention involved a mentoring circle of experienced nutritionists and community-based dieticians. Each participant attended six 2-h sessions every 6 weeks for a 7-month intervention period	Nutrition knowledge and competence
Lindorff-Larsen et al. (17), Denmark	Pre–post-intervention study	A follow-up study was conducted in 2004 and compared to a baseline study in 1997. Nutrition training and use of nutrition guidelines were being introduced and used between the two study intervals. Details of such training were not further elaborated	Nutrition knowledge
Puoane et al. (38), South Africa	Pre–post-intervention study (with a qualitative design)	A 5-day course developed by the University of West Cape involved practice sessions, group work, role-plays, action plan development, key messages, and question and answer sessions. The course followed the principle of care set out by WHO for managing severe malnutrition. A total of 66 nurses from 11 referral hospitals underwent this course	Health workers’ practice Attitudes
Hamer et al. (39), Gambia	Pre–post-intervention study	Nutrition training for nurses was conducted using the IMCI training manual, “assess and classify sick children aged 2 months to 5 years.” Training materials were provided to nurses a week prior to training. The training included both theoretical and practical components toward assessing children with and without wasting and/or edema admitted to the hospital	Nutrition knowledge Undernutrition management/practice
Edwards and Wyles (40), UK	Pre–post-intervention study	A total of 24 1-h training sessions were held, reaching 189 staff. Each session consisted of factual information, a brainstorming session about what a pregnant woman eat, and a nutrition game involving calculation of daily requirement for folic acid	Nutrition knowledge Health workers’ practice
O’Mahony et al. (41), UK	Pre–post-intervention study	Nutrition training was delivered to nurse participants. It also included the use of the Malnutrition Universal Screening Tool (MUST)	Nutrition knowledge Health workers’ practice
Hillenbrand and Larsen (20), US	Pre–post-intervention study	Forty-nine pediatric residents participated in a four-part education series about breastfeeding delivered over four consecutive days. The education intervention was internally designed using additional inputs from lactation experts and fellow pediatricians. The training included lectures, discussions, role-playing, and group exercises	Nutrition knowledge Nutrition counseling Counseling and practice
Olsson et al. (42), Sweden	Pre–post-intervention study	Nutrition education for nurses was conducted for 3 months. It was based on the use of nutrition assessments including energy intake, clinical complications of inadequate energy intake, hospital food energy content, patients energy requirements, weighing patients and its necessity, reasons for weight loss during illness, and fluid management	Nutrition knowledge Health workers’ practice
Pedersen et al. (43), Denmark	Pre–post-intervention study	Nutrition training was conducted for nurses. It included five modules of 3–4 days duration using the theory of planned change. The training incorporated basic nutrition education elements such as risk assessment, consequences of malnutrition, and assessment of needs and responsibility	Health workers’ practice
Gance-Cleverland et al. (21), US	Pre–post-intervention study	Thirty-five nurse practitioners received an intensive 4-h Healthy Eating and Activity Together Clinical Practice Guideline (HEAT CPG) training session	Nutrition knowledge Nutrition counseling
Bjerrum et al. (18), Denmark	Pre–post-intervention study	Sixteen nurses participated in a special training program on nutrition. It was based on experimental theories and included five modules spanning 3–4 days, combining theories of planned change and nutrition issues	Nutrition knowledge
Kennelly et al. (45), Ireland	Pre–post-intervention study	Seven general practitioners participated in the nutrition education program. A community dietician used a standardized presentation to conduct the program. The content of training included information on causes of malnutrition, effects of malnutrition, use of the MUST tool, practical dietary advice for patients with poor appetite, and evidence supporting the use of oral nutrition supplements (ONS)	Nutrition counseling Health workers’ practice
Kennelly et al. (44), Ireland	Pre–post-intervention study	An educational program incorporating the MUST training was implemented in 8 of 10 eligible primary practices, 7 private nursing homes, and 2 health centers. The training program was designed based on consultations with health professional groups, clinical guidelines from expert bodies, and current evidence for ONS use in community settings	Nutrition knowledge Nutrition counseling
Simoes et al. (46), Ethiopia	Pre–post-intervention study	A 9-day course using the pre-tested version of the IMCI course was provided to six clinic nurses. The training modules included assessment and classification of the sick child, treatment of the child, counseling the mother, and follow-up. Other modules included practical sessions in the clinic	Nutrition knowledge Nutrition counseling Health workers’ practice
Davies-Adetugbo and Adebawa (47), Nigeria	Pre–post-intervention study	A 1-day community mobilization with 6 h of training on breastfeeding and child survival was conducted for health workers and mothers. The training was designed to include the importance of breast-feeding, exclusive breastfeeding, lactation maintenance, expressed breast milk, practical demonstration of attachment, suckling, expression of milk, and cup feeding. An intensive 2-day training was then conducted for health workers using a WHO/UNICEF 18-h breastfeeding course manual. Training included practical, role-playing, and theory sessions	Nutrition and breastfeeding knowledge
Newes-Adeyi et al. (48), US	Pre–post-intervention study	A total of 35 women, infants, and children (WIC) staff underwent a 1-day intensive training program to improve their growth monitoring counseling and management of nutrition-related problems. The training included lectures, case studies, discussions, small group work, and role-plays	Nutrition counseling
Stark et al. (49), US	Quasi-experimental design	A 6-week online professional development program for nutrition and health practitioners course was delivered to the intervention group. It was based on the PRECEDE-PROCEED health program planning framework involving assessment of underlying factors for a health problem and strategizing the intervention	Nutrition knowledge and skills
Charlton et al. (24), Zambia	Pre–post-intervention study	Eight out of 16 health care workers received the Growth Monitoring and Promotion (GMP) training. Details of the training including duration and contents were not described	Nutrition knowledge Nutrition practice

Nutrition training spanned varying forms and duration. The most common standard used was the IMCI model, based on the “counsel the mother” and “nutrition-counseling” modules (15, 19, 22, 39, 46). Other training frameworks included the WHO standard counseling training modules (13, 16, 38, 47), tailored nutrition training using the Malnutrition Universal Screening Tool (MUST) (41, 44, 45), and nutrition training prepared specifically to suit the specific situation or context. Across training types, the lowest training duration was 4 h (21), while the longest training duration was 6 weeks of online training (49).

The main outcome of interest from the selected studies are presented in Table 4 according to our objectives. Health workers’ nutrition knowledge after the nutrition training intervention was reported in a total of 18 studies: 3 RCTs (13–15), 1 controlled non-randomized trial (16), and 14 pre–post-intervention studies (17, 18, 20, 21, 37, 39–42, 44, 46, 47). Counseling and communication skills of health workers who underwent the nutrition intervention were reported in 12 studies: 6 RCTs (13–15, 19, 22, 23) and 6 pre–post-intervention studies (20, 21, 44–46, 48). A total of 16 studies reported health workers’ management skills for child undernutrition after undergoing nutrition training. Among such studies, 3 were RCTs (13, 15, 22), 1 was a controlled non-randomized trial (16), and 14 were pre–post-intervention evaluation studies (24, 37–46, 49).

### Effectiveness of the intervention to improve health workers’ nutrition knowledge (a narrative summary)

Table 5 summarizes the 18 reviewed studies that included nutrition knowledge as the outcome variable. Among these studies, three used a cluster RCT design and were conducted in Brazil (13, 15) and the UK (14). A higher proportion of doctors who received the nutrition training intervention in the Brazilian studies had high post-training nutrition knowledge compared to doctors in the control group. In the UK-based RCT among GPs, nutrition training did not significantly change health workers’ nutrition knowledge (14). However, GPs in the intervention group were 30% more likely to believe that their nutrition knowledge was up-to-date compared to their counterparts in the control group (*P* = 0.001).

**Table 5:** **The effectiveness of nutrition training to improve nutrition knowledge of health care workers**.

Author	Study design	Health cadre	Nutrition training intervention	Comparison	Outcome: nutrition knowledge
Bassichetto and Réa (13), Brazil	RCT: 31 professionals received intervention and 28 were the control	Pediatricians and nutritionists	WHO’s “infant and young child feeding counseling: an integrated course.” The training includes 8 h of practical sessions. Out of 34 sessions, 8 were dedicated to breastfeeding, 6 to HIV and infant and young child feeding, 7 to complementary feeding, 10 to counseling, and 4 to general themes	Doctors and nutritionists in control group did not receive the training intervention.	Proportion of knowledge increase was more among HCWs in IG [e.g., Breastfeeding – IG-79.3%, CG-37% (*P* = 0.004); HIV and IYCF – IG-48.3%, CG-18.5% (*P* = 0.049); Complementary feeding – IG-69.0%, CG-37.0% (*P* = 0.012)]
Moore et al. (14), UK	Cluster RCT-paired cluster randomized trial with pre- and post-intervention assessment	12 General practitioners	A training program was delivered to six intervention practices. Emphasis was on increasing motivation to improve quality of dietary consultations and providing practical skills adapted from behavior models. A 7.5-h training included patients’ assessment, education, and goal setting in issues of public health importance including drinking	Six control practices did not receive nutrition training	IG-trained practitioners were 30% (95% CI 12–50, *P* = 0.001) more likely to believe that their knowledge was up-to-date than practitioners in IG. There was no statistical significance difference in actual knowledge between IG and CG
Santos et al. (15), Brazil	RCT of 28 government health centers	28 Medical doctors	A total of 14 doctors in the intervention group received a 20-h nutrition-counseling training and practice using IMCI’s “counsel the mother” and “management of the sick young infant” modules. The key recommendations identified were as follows: increase breast and complementary feeding frequency, provide animal protein and micronutrient-rich foods, add oil to the food, and increase dietary diversity	14 Doctors recruited for CG did not receive training	Doctors from IG correctly answered 83% (95% CI 65–100) of 77 questions on practical situations in the IMCI guidelines compared to 68% (95% CI 48–88) in the CG (*P* = 0.02)
Cattaneo and Buzzetti (16), Italy	Controlled non-randomized study	Nurses, midwives, obstetricians, and physicians	An 18-h UNICEF “breastfeeding, management, and health proportion in baby-friendly hospitals” course along with a 2-h counseling session from the WHO breast-feeding counseling course were implemented	Post-training evaluation	In Group 1, nutrition knowledge went up from a mean score of 0.41 to 0.66 to 0.72. In Group 2, nutrition knowledge went from 0.53 to 0.53 to 0.75
Palermo et al. (37), Australia	Pre–post-intervention study	Nutritionists and dieticians	A total of 32 dieticians were allocated to three IGs: two face-to-face groups and one rural video-linked group. The intervention involved a mentoring circle of experienced nutritionists and community-based dieticians. Each participant attended six 2-h sessions every 6 weeks for a 7-month intervention period	Pre–post-intervention comparison (qualitative and quantitative)	Reported competency score increased post-training/mentoring. An increase in post-intervention measures was also reported: [69.1(13.8) to 79.3(12.1), *P* < 0.001]
Lindorff-Larsen et al. (17), Denmark	Pre–post-intervention study	Doctors and nurses	A follow-up study was conducted in 2004 and compared to a baseline study in 1997. Nutrition training and guidelines were being introduced and used between the two study intervals. Details of such training were not further elaborated	A cross-sectional study, post-trainings and post-guideline application	About two-thirds of doctors and nurses expressed that their education nutrition was sufficient at post-intervention. Significantly fewer health workers lacked methods to identify undernutrition (*P* < 0.001) and difficult-to-identify patients in need of nutrition support (*P* < 0.001) at post-intervention
Hamer et al. (39), Gambia	Pre–post-intervention study	Registered nurses and auxiliary nurses	Nutrition training for nurses was conducted using the IMCI training manual, “assess and classify sick children aged 2 months to 5 years.” It included both theoretical and practical components of assessing children with and without wasting and edema	Post-training evaluation	Nurses showed good knowledge and performance after the completion of training
Edwards and Wyles (40), UK	Pre–post-intervention study	Midwives, physicians, dieticians, and nurses	A total of 24 1-h training sessions were held for 189 staff. Each session consisted of factual and brainstorming sessions about what a pregnant woman eats, and a nutrition calculation of daily requirement for folic acid	Post-training evaluation	Health workers’ nutrition knowledge improved post-training
O’Mahony et al. (41), UK	Pre–post-intervention study	Nursing staff	Nutrition training was delivered to nurse participants on the use of the Malnutrition Universal Screening Tool (MUST)	Post-training evaluation	A non-significant difference in post-training nutrition knowledge was observed [Mean (SD) knowledge score 21(6.7) vs. 23(6.2)]. A significant difference was observed in sub-analyses by bands. Nurses were more aware that malnutrition was a significant problem for the National Health Service post-training (*P* < 0.027)
Hillenbrand and Larsen (20), US	Pre–post-intervention study	Pediatric residents	A total of 49 pediatric residents participated in a four-part education series about breastfeeding over 4 consecutive days. It included lectures, discussions, role-playing, and group exercises. The education intervention was internally designed by the authors using inputs from lactation experts and fellow pediatricians	Post-training evaluation	Mean composite knowledge score was 80% post intervention compared to 69% pre-intervention, representing an 11% increase (*P* < 0.01)
Olsson et al. (42), Sweden	Pre–post-intervention study	Nurses	Nutrition education for nurses was conducted for 3 months. It was based on the use of nutrition assessment including energy intake, clinical complications of inadequate energy intake, hospital food energy, patients’ energy requirements, weighing patients and its necessity, reasons for weight loss during illness, and fluid management	Post-training evaluation	69% Of nurses could calculate a patient’s energy requirement post-training compared to 24% pre-training (*P* < 0.01). Compared to pre-training, more nurses knew the energy content of hospital food (61 vs. 45%, *P* < 0.05), knew how to handle enteral infusion equipment (55 vs. 6%, *P* < 0.01), and found it easy to assess patients’ energy needs (56 vs. 24%, *P* < 0.01)
Gance-Cleverland et al. (21), US	Pre–post-intervention study	Nurse practitioners	A total of 35 nurse practitioners received an intensive 4-h Healthy Eating and Activity Together Clinical Practice Guideline (HEAT CPG) training session	Post-training evaluation	Nutrition knowledge post training improved, including on assessment of growth (*P* < 0.001), assessment of family history (*P* < 0.001), and assessment of physical activity (*P* < 0.001). Practitioners’ nutrition recommendation knowledge also improved post-training compared to pre-training
Bjerrum et al. (18), Denmark	Pre–post-intervention study	Nurses	A total of 16 nurses participated in a special training program on nutrition. It was based on experimental theories. A total of five modules lasting 3–4 days were included. They combined theories of planned change and nutrition issues	Post-training evaluation	A short-duration training program enhanced nurses’ awareness of nutrition care, management through assessment and monitoring, their management roles, and approach to clinical nutrition
Kennelly et al. (44), Ireland	Pre–post-intervention study	General practitioners (GPs) and nurse practitioners	An educational program incorporating Malnutrition Universal Screening Tool (MUST) training was implemented in 8 of 10 eligible primary practices, seven private nursing homes, and two health centers. The training program was designed based on consultations with health professional groups, clinical guidelines from expert bodies, and current evidence for oral nutrition supplementation (ONS) use in community settings	Post-training evaluation	Nutrition knowledge improved across three evaluation points (*P* < 0.05). For specific groups, a significant improvement in knowledge score was also observed among general practitioners (*P* < 0.001) and nurses (*P* < 0.001)
Simoes et al. (46), Ethiopia	Pre–post-intervention study	Clinic nurses	Six clinic nurses received a 9-day course using the pre-tested version of the IMCI course. The training modules included assessment and classification of a sick child, treatment of the child, counseling the mother, and follow-up. Other modules included practical sessions in the clinic	Post-training evaluation	After training, nurses could recognize visible severe wasting with a 67% sensitivity and 99% specify; conjunctiva pallor for anemia at 45% sensitivity and 94% specificity; and bipedal edema with 69% sensitivity and 98% specificity
Davies-Adetugbo and Adebawa (47), Nigeria	Pre–post-intervention study	Community health extension workers	A 6-h training on breastfeeding and child survival was conducted for health workers and mothers. The training included the importance of breast-feeding, exclusive breastfeeding, lactation maintenance, expressed breast milk, practical demonstration of attachment, suckling, expression of milk, and cup feeding. An intensive 2-day training was then conducted for health workers using a WHO/UNICEF 18-h breastfeeding course manual. Training included practical, role-playing, and theory sessions	Post-training evaluation	Trained health workers had a significantly higher aggregate knowledge score compared to their untrained counterparts [9.4(9.1–9.7) vs. 7.6(6.6–8.6), *P* < 0.001]
Stark et al. (49), US	Quasi-experimental design using intervention and delayed intervention comparison group	Nutrition and health professionals	An online professional development program for nutrition and health practitioners course was given to the intervention group for 6 weeks. It was based on the PRECEDE-PROCEED health program planning framework involving assessment of underlying factors for a health problem and strategizing the intervention	Delayed intervention control group	Compared to the control group, the intervention group reported significant positive changes (*P* < 0.01) on knowledge and skills scores
Charlton et al. (24), Zambia	Pre–post-intervention study	Health workers for growth monitoring and promotion	Eight out of 16 HCWs received the growth monitoring and promotion training	Post-training evaluation	Compared to untrained HCWs, trained HCWs could correctly define growth monitoring and promotion (*P* < 0.001)

IG, intervention group; CG, control group; HCWs, health care workers.

In an Italian controlled non-randomized trial with a comparison group (16), the mean nutrition knowledge score of nurses, midwives, and doctors increased from 0.41 to 0.72 in Group 1 and from 0.53 to 0.75 in Group 2 after the nutrition training. Compared to the delayed intervention control group, health workers in the intervention group registered a significant change in knowledge and skills (*P* < 0.01) in a quasi-experimental study conducted in the US.

Fourteen studies were conducted using a pre–post-intervention design in Australia (37), Denmark (17, 18), Gambia (39), UK (41), US (20, 21, 49), Sweden (42), Ireland (44), Ethiopia (46), Nigeria (47), and Zambia (24). In all of these studies, health workers’ nutrition knowledge increased after nutrition training.

### Effectiveness of intervention to improve nutrition-counseling skills of health workers (a narrative summary)

Table 6 shows the result of the 12 reviewed studies with nutrition counseling as an outcome variable following nutrition training of health workers. Of these studies, six were cluster RCTs and three were conducted in Brazil among doctors and pediatricians (13, 15, 19). Across all three studies, a significantly higher proportion of doctors in the intervention group had better post-training counseling skills and performed more nutrition counseling compared to the respective control group. Physicians undergoing the training intervention also showed higher mean communication skill scores compared to untrained physicians (*P* < 0.01) (19). In a Pakistani cluster RCT among lady health visitors (LHVs) working at a health facility as mid-level providers, 82% of participants in the intervention group registered improved post-training communication skills compared to 51% in the control group (*P* = 0.015). In this study, a higher proportion of trained LHVs reported increased counseling skills compared to their counterparts. GPs with nutrition training in the UK study (14), meanwhile, were 30% more likely to provide dietary advice that was completely appropriate (*P* = 0.01). In Peru (23), twice as many mothers in the intervention group received post-partum nutrition advice compared to their control group counterparts following the nutrition training intervention for their health workers (*P* = 0.02).

**Table 6:** **Effectiveness of nutrition training to improve nutrition counseling and counseling skills of caregivers**.

Author	Study design	Health cadre	Intervention	Comparison	Outcome: nutrition counseling
Zaman et al. (22), Pakistan	Cluster RCT: 18 health centers were assigned to IG and a similar number to CG	Lady health visitors (MLVs)	Nutrition-counseling training using IMCI’s “counsel the mother” module for five and a half days. It included infant feeding knowledge and practice sessions to develop communication and counseling skills	Health centers of the control group without counseling training for health workers	Counseling: asking about feeding practices and paying attention to answers: IG-50%, CG-25%, *P* = 0.056; praising mothers for positive action: IG-37%, CG-8%, *P* = 0.006. Appropriate recommendations to specific changes with explanation: IG-29%, CG-4%, *P* = 0.01
					Communication skills: IG-82%, CG-51%, *P* = 0.015
Bassichetto and Réa (13), Brazil	RCT: 31 professionals received intervention and 28 were recruited as a control group	Pediatricians and nutritionists	WHO’s “Infant and young child feeding counseling: an integrated course” was administered. The training includes 8 h of practical sessions. Out of 34 sessions, 8 were dedicated to breastfeeding, 6 to HIV and infant and young child feeding, 7 to complementary feeding, 10 to counseling, and 4 to general themes	Participants recruited for the control group did not receive the training intervention	Counseling: IG-51.7%, CG-22.2% (*P* = 0.004)
Moore et al. (14), UK	Cluster RCT-paired cluster randomized trial with pre- and post-intervention evaluation	General practitioners	A training program was delivered to six intervention practices. Emphasis was on increasing motivation to improve quality of dietary consultations and providing practical skills adapted from behavior models. A 7.5-h training included patients’ assessment, education, and goal setting in issues of public health importance including drinking	A total of six control practices did not receive nutrition training	Counseling: trained practitioners were 30% (95% CI 7–53, *P* = 0.01) more likely to provide dietary advise that was completely appropriate
Pelto et al. (19), Brazil	Cluster RCT of 28 municipal health centers	Doctors	Physicians from the intervention group received a 20-h training in a program derived from the IMCI nutrition-counseling module. After training, they provided care to caregiver/child pairs attending their centers	Physicians in the control group received a clinical refresher course but not on nutrition counseling	Counseling: trained providers engaged more in nutrition counseling [only 9(24%) consultations of IG participants did not include advice compared to 14 (43%) among CG participants: *P* < 0.013]; gave 81 messages compared to 20 of untrained ones (*P* < 0.002); gave more message specific to foods, preparations, and feeding practices compared to untrained ones (*P* < 0.01)
					Communication skills: mean communication skills score of trained physicians was 3.94 (SD 1.68) vs. 1.38 (SD 1.02) for untrained ones (*P* < 0.01)
Santos et al. (15), Brazil	RCT of 28 government health centers	28 Medical doctors	A total of 14 doctors of the IG received a 20-h nutrition-counseling training and practice using IMCI’s “counsel the mother” and “management of the sick young infant” modules. The key recommendations identified were as follows: increase breast and complementary feeding frequency, give animal protein and micronutrient-rich foods, add oil to the food, and increase dietary diversity	14 doctors recruited for the control group did not receive the training	Counseling: 83% of mothers in IG compared to 49% of mothers in CG received nutrition counseling (*P* < 0.001)
Penny et al. (23), Peru	Cluster RCT of 12 health facilities serving periurban areas	Health workers in selected health facilities	The intervention included training for HCWs to improve anthropometry skills. An accreditation system was also introduced for institutional change. Also it included demonstration of preparation of complementary foods and child’s age-specific group sessions for caregivers	HCWs and caregivers of CG did not receive the training intervention	Counseling: twice as many mothers in IG received nutrition advice after birth compared to those in CG (52 vs. 24%, *P* = 0.02). Greater impacts on counseling were observed at 4 and 18 months post-intervention (*P* < 0.002)
Hillenbrand and Larsen (20), US	Pre–post-intervention study	Pediatric residents	A total of 49 pediatric residents participated in a four-part education series about breastfeeding over four consecutive days. The training included lectures, discussions, role-playing, and group exercises. The education intervention was designed using additional inputs from lactation experts and fellow pediatricians	Post-training evaluation	Counseling: residents showed an increased knowledge in advising mothers concerning low milk supply (*P* = 0.045), infections including mastitis (*P* = 0.002), or abscess (*P* < 0.001)
					Counseling and practice: residents showed significant increases in counseling on signs of breast-feeding adequacy (*P* = 0.012) and managing lactation problems correctly (*P* = 0.004)
Gance-Cleverland et al. (21), US	Pre–post-intervention study	Nurse practitioners	A total of 35 nurse practitioners received an intensive 4-h Healthy Eating and Activity Together Clinical Practice Guideline (HEAT CPG) training session	Post-training evaluation	Counseling: participants reported a significant improvement in behavior modification techniques (*P* < 0.001) and practitioners’ counseling (*P* < 0.001)
Kennelly et al. (45), Ireland	Pre–post-intervention study	General practitioners-doctors	Seven GPs participated in the nutrition education program. The content of training included causes of malnutrition, effects of malnutrition, the use of the Malnutrition Universal Screening Tool (MUST), practical dietary advice for patients with poor appetite, and evidence supporting the use of oral nutrition supplements (ONS)	Post-training evaluation	Counseling: basic dietary advice provided by a health professional increased significantly post-training (90 vs. 26%, *P* < 0.001)
Kennelly et al. (44), Ireland	Pre–post-intervention study	General practitioners (GP) and nurse practitioners	An educational program incorporating the MUST training was implemented in 8 of 10 eligible primary practices, seven private nursing homes, and two health centers. The training program was designed based on consultations with health professional groups, clinical guidelines from expert bodies, and current evidence for ONS use	Post-training evaluation	Counseling: about 80% of HCWs reported always providing nutrition advice to patients
Simoes et al. (46), Ethiopia	Pre–post-intervention study	Clinic nurses	A 9-day course using the pre-tested version of the IMCI course was provided to six clinic nurses. The training modules included assessment and classification of sick child, treatment of the child, counseling the mother, and follow-up. Other modules included practical sessions in the clinic	Post-training evaluation	Counseling: trained health workers provided feeding advice rated as “good” by 78%, “fair” at 18% and “poor” at 4%
Newes-Adeyi et al. (48), US	Pre–post-intervention study	Health workers of a special nutrition program	A total of 35 health workers underwent a 1-day intensive training program to improve their growth monitoring counseling and management of nutrition-related problems. The training included lectures, case studies, discussions, small group work, and role-plays	Post-training evaluation	Counseling: compared to pre-training, there was a significant change in elicitation (*P* < 0.001) and negotiation proficiency (*P* = 0.07). The level of engagement in discussing provider suggestions for follow-up strategies increased from 1.8 to 2.3 (*P* < 0.01) and the overall responsiveness level increased from a mean of 2.4–2.8 (*P* < 0.07)

IG, intervention group; CG, control group; HCWs, health care workers.

A total of six studies using pre–post-intervention evaluation of nutrition training of health workers reported nutrition-counseling skills as an outcome variable. These studies were conducted in the US (20, 21, 48), Ireland (44, 45), and Ethiopia (46). In all six studies, nutrition and general counseling skills of health workers improved after nutrition training.

### Effectiveness of intervention to improve health workers’ management practices for child undernutrition (a narrative summary)

Table 7 summarizes the results of the 16 reviewed studies reporting management of undernutrition and management practices as an outcome variables following the nutrition training intervention. Within these studies, two of the three cluster RCTs were conducted in Brazil among medical doctors, pediatricians, and nutritionists (13, 15). Doctors in the intervention group were more likely to report improved post-intervention practices in managing child undernutrition compared to their counterparts. In the Pakistani study, trained LHVs were more likely to plot children’s weights, discuss appropriate foods with caregivers, and check mothers’ understanding of imparted nutrition knowledge compared to their counterparts in the control group. In an Italian controlled non-randomized trial (16), all hospitals improved their compliance with WHO’s “Ten Steps to Successful Breastfeeding” after undergoing the WHO baby-friendly hospital and counseling course.

**Table 7:** **Effectiveness of nutrition training to improve nutrition management practices and competence of health workers**.

Author	Study design	Health cadre	Intervention	Comparison	Outcome: nutrition management or practice
Zaman et al. (22), Pakistan	Cluster RCT: 18 health centers were assigned IG and a similar number were assigned to CG	Lady health visitors	Nutrition-counseling training using IMCI’s “counsel the mother” module for five and a half days. It included infant feeding knowledge and practice sessions for development of communication and counseling skills	HCWs of the CG received no counseling training	Practice: HCWs in the intervention group were more likely to plot the weight of a child, discuss foods appropriate to the child, and check if mothers understood information provided
Bassichetto and Réa (13), Brazil	RCT: 31 professionals recruited to IG and 28 for CG	Pediatricians and nutritionists	WHO’s “infant and young child feeding counseling: an integrated course” was implemented. The training includes 8 h of practical sessions. Out of 34 sessions, 8 were dedicated to breastfeeding, 6 to HIV and infant and young child feeding, 7 to complementary feeding, 10 to counseling, and 4 to general themes	HCWs in the CG did not receive the training intervention	Performance: IG participants improved their dietary anamnesis during consultations after intervention (*P* < 0.001)
Santos et al. (15), Brazil	RCT of 28 government health centers assigned to either IG or CG	28 Medical doctors	A total of 14 doctors of IG received a 20-h nutrition-counseling training and practice using IMCI’s “counsel the mother” and “management of the sick young infant” modules. The key recommendations identified were as follows: increase breast and complementary feeding frequency, provide animal protein and micronutrient-rich foods, add oil to the food, and increase dietary diversity	Doctors in the CG did not receive counseling training	Practice: doctors from IG were more likely to assess child’s complementary feeding, assess breast-feeding, use good communication skills, and use and provide mothers with a card compared to CG
Cattaneo and Buzzetti (16), Italy	Controlled non-randomized	Nurses, midwives, obstetricians, and physicians	An 18-h UNICEF “breastfeeding, management, and health proportion in baby-friendly hospitals” course along with a 2-h counseling session from the WHO breast-feeding counseling course was implemented	Post-training evaluation	Performance: all hospitals improved their compliance with the WHO 10 steps to successful breastfeeding
Palermo et al. (37), Australia	Pre–post-intervention study	Nutritionists and dieticians	A total of 32 dieticians were allocated to three intervention groups: two face-to-face groups and one rural video-linked group. The intervention involved a mentoring circle of experienced nutritionists and community-based dieticians. Each participant attended six 2-h sessions every 6 weeks for a 7-month intervention period	Pre–post-intervention comparison (qualitative and quantitative)	Nutrition competence: reported competency scores increased post training/mentoring. An increase in post-intervention measures was also observed: [69.1(13.8) to 79.3(12.1), *P* < 0.001]
Puoane et al. (38), South Africa	Pre–post-intervention study (with a qualitative design)	Nurses	A 5-days course developed by the University of West Cape was administered. It involved practice sessions, group work, role-plays, development of an action plan, key messages, and question and answer sessions. The course followed the principle of care set out by WHO for managing severe malnutrition	Post-intervention (training)	Practice: in-patient care for malnutrition management improved after the training. This included adequate follow-up on the 10 steps to management of malnutrition
Hamer et al. (39), Gambia	Pre–post-intervention study	Registered and auxiliary nurses	Nutrition training for nurses was conducted using the IMCI training manual, “Assess and classify sick children aged 2 months to 5 years.” It included both theoretical and practical components of assessing children with and without wasting and/or edema admitted to the hospital	Post-training evaluation	Practice: in assessing undernutrition, nurses showed a 56% sensitivity, 95% specificity, and 56% positive predictive value (PPV)
Edwards and Wyles (40), UK	Pre–post-intervention study	Midwives, physicians, dieticians, and nurses	A total of 24 1-h training sessions were held, reaching 189 staff. Each session consisted of factual information, a brainstorming session about what a pregnant woman eats, and a nutrition game involving calculation of daily requirement for folic acid	Post-training evaluation	Practice: in a nutrition game, a high average intake of folic acid was observed in the chosen food items. It ranged from 244 to 500 μg compared to only 219 μg shown in average in census data on the same population
O’Mahony et al. (41), UK	Pre–post-intervention study	Nursing staff	Nutrition training was conducted with nurse participants. It also included the use of the Malnutrition Universal Screening Tool (MUST)	Post-training evaluation	Practice: 94% of nurses weighed patients on admission post-training compared to 74% before (*P* < 0.001)
Olsson et al. (42), Sweden	Pre–post-intervention study	Nurses	Nutrition education for nurses was conducted for 3 months. Training was based on the use of nutrition assessments including energy intake, clinical complication of inadequate energy intake, hospital food energy content, patients’ energy requirements, weighing patients and its necessity, reasons for weight loss during illness, and fluid management	Post-training evaluation	Practice: compared to pre-training, during post-training, nurses were more likely to use food forms to document food intake (*P* < 0.01)
Pedersen et al. (43), Denmark	Pre–post-intervention study	Nurses	Nutrition training was conducted for nurses. It included five modules spanning 3–4 days using the theory of planned change. The intervention involved basic nutrition education elements such as risk assessment, consequences of malnutrition, and assessment of needs and responsibility	Post-training evaluation	Practice: after the training, more patients reported eating difficulties to staff (*P* = 0.01), none reported not receiving help in cutting their food (*P* = 0.014), fewer had difficulty in chewing (*P* = 0.01), and fewer reported not receiving food they did not order (*P* = 0.01)
Kennelly et al. (45), Ireland	Pre–post-intervention study	General practitioners-doctors	Seven general practitioners participated in the nutrition education program. A community dietician used a standardized presentation to conduct the program. The content of training included information on causes of malnutrition, effects of malnutrition, the use of MUST, practical dietary advise to patients with poor appetite, and evidence supporting the use of oral nutrition supplements (ONS)	Post-training evaluation	Practice: about 62% completed a nutrition screening tool (MUST) on referral to a community dietician compared to 0% pre-intervention (*P* < 0.001). A greater proportion of patients with high risk of malnutrition were prescribed ONS post-training compared to pre-training (88% vs. 37%, *P* < 0.001)
Kennelly et al. (44), Ireland	Pre–post-intervention study	General practitioners (GP) and nurse practitioners	An educational program incorporating MUST training was implemented in 8 of 10 eligible primary practices, 7 private nursing homes, and 2 health centers. The training program was designed based on consultations with health professional groups, clinical guidelines from expert bodies, and current evidence for ONS use in community settings	Post-training evaluation	Practice: management of malnutrition improved post training. About 69% of HCWs weighed patients more frequently and 80% reported on the usefulness of MUST
Simoes et al. (46), Ethiopia	Pre–post-intervention study	Clinic nurses	A 9-day course using the pre-tested version of the IMCI course was provided to six clinic nurses. The training modules included assessment and classification of a sick child, treatment of the child, counseling the mother, and follow-up. Other modules included practical sessions in the clinic	Post-training evaluation	Practice: compared to pediatricians, the trained nurses could diagnose malnutrition and anemia classified as severe or some malnutrition at a sensitivity of 85% and specificity of 96%
Stark et al. (49), US	Quasi-experimental design	Nutrition and health professionals	An online professional development program for nutrition and health practitioners course was given to the intervention group for 6 weeks. It was based on the PRECEDE-PROCEED health program planning framework involving assessing underlying factors for a health problem and strategizing the intervention	Delayed intervention control group	Nutrition management skills: compared to the control group, the intervention group reported positive changes (*P* < 0.01) on knowledge and skills scores
Charlton et al. (24), Zambia	Pre–post-intervention study	Health workers of growth monitoring and promotion	Eight out of 16 HCWs received the Growth Monitoring and Promotion (GMP) training. Details of the training including duration and contents were not explained	Post-training evaluation	Practice: trained HCWs could correctly interpret growth cards and complete the under-five card compared to their untrained counterparts (*P* < 0.05)

IG, intervention group; CG, control group; HCWs, health care workers.

Nutrition and health professionals in the quasi-experimental design study in the US exhibited better nutrition management skills after the 6-week online nutrition training (49). Compared to health workers in the delayed intervention control group, those in the intervention group registered significant positive changes on knowledge and skills scores (*P* < 0.01).

Eleven pre–post-intervention studies on nutrition training of health workers were conducted in Australia (37), South Africa (38), Gambia (39), UK (40, 41), Sweden (42), Denmark (43), Ireland (44, 45), Ethiopia (46), US, and Zambia (24). In all these studies, management practices and competence of health workers improved after the intervention compared to pre-nutrition training intervention levels.

## Discussion

This is the first systematic review to examine the effectiveness of in-service nutrition training to improve health workers’ nutrition knowledge, nutrition counseling, and undernutrition management practices. In this review, we reviewed a total of 25 studies reporting on nutrition training interventions. Across all three of our outcome variables, significant post-intervention improvements were reported. First, in-service nutrition training improved health workers’ nutrition knowledge. Second, the counseling skills and competence of health workers were also improved after in-service nutrition training. Third, the training intervention improved child undernutrition management practices of participating health workers.

A total of 18 studies, including five with a cluster RCT design, showed significant post-nutrition training improvements in health workers’ nutrition knowledge. These studies were conducted in areas of varying social and economic levels and geographic characteristics. Health workers might also have been exposed to nutrition education during their college training (8). However, previous studies have indicated that such training is inadequate or not in keeping with the clinical reality encountered in practice (9, 10). Lack of such knowledge might also cause them to refrain from providing nutrition counseling and care to their clients (8). Sometimes, due to lack of adequate nutrition knowledge, doctors feel that it is the duty of nurses or other cadres below them to provide nutrition counseling and care. To improve knowledge of such health workers, it is important to expose them to in-service nutrition training tailored to their environment, context, and health cadre (9). This will help to boost their competence and confidence in management of nutrition-related conditions including undernutrition.

In this systematic review, a total of 12 studies, including six cluster RCTs, showed a significant improvement in counseling skills among health workers with in-service nutrition training. Nutrition training would thus seem to be effective in improving health workers’ nutrition knowledge. Nutritionally informed health workers may be more confident to address nutrition-related conditions in their patients (51). Such health workers may be better equipped to provide appropriate advice and counseling to their clients. The prevailing attitude toward nutrition counseling among medical doctors and pediatricians that such functions are not within their job description might also change after nutrition training. In this way, such trained health workers would be more likely to provide nutrition counseling (52). In line with the health belief model, nutrition knowledge provided to the health workers through nutrition training is more likely to influence counseling behavior (27). Further, according to Bandura’s social learning theory, such behavior or attitude change is mediated through cognitive processes and thus is learned through imitating and observing the actions of others (27). Accordingly, the reward that health workers can gain from their nutrition-counseling actions, such as better nutritional status or feeding practices in those they treat, may reinforce their counseling actions, thus making it a permanent habit. In this way, the quality of health workers with regard to nutrition counseling might be expected to improve.

Our review showed that health workers’ undernutrition management practices improved when they received in-service nutrition training. A total of 16 intervention studies, including three cluster RCTs, showed a significant improvement in management practices for child undernutrition after nutrition training. Barriers to effective management of child undernutrition include lack of nutrition knowledge and counseling skills among health providers (12). Such barriers can be ameliorated when health workers receive appropriate and tailored in-service nutrition training suited to their context and cadre.

Findings from this review should be carefully considered in the context of two primary limitations. First, the results are not based on meta-analysis to calculate the overall effect size of the intervention for each outcome variables. This was due to variations in the study designs and measurements used for outcome variables, and to differences in the competence, experience, and cadres of participating health workers. Such variations could have resulted in high heterogeneity. Hence, instead of meta-analyses, we explained each study separately in a narrative summary stratified by outcome variable. Although we did not pool our results, individual studies showed a significant effect of nutrition interventions on outcome variables.

Second, the included studies differed in the intervention’s length and content. This might have caused differences in the measured outcome variables. However, most of the studies used standard nutrition training frameworks for health workers including the IMCI nutrition-counseling training module, breast-feeding counseling training modules by both WHO and UNICEF, MUST training modules, and other comparable training based on formative research. Despite such differences, each study showed a significant improvement in one or more of the outcome variables.

Despite its limitation, findings thus presented may help decision makers to plan and conduct in-service nutrition training for health workers, an important building block toward building a strong foundation for any health system. This review is the continuation of series papers on the effectiveness of nutrition training of health workers. It is the first systematic review on the effectiveness of nutrition training for health workers on their nutrition knowledge, nutrition counseling, and undernutrition management skills. The other paper in the series found that, nutrition training of health workers improved feeding frequency, energy intake, and dietary diversity of children aged 6 months to 2 years (28).

In conclusion, in-service nutrition training of health workers improves their nutrition knowledge, nutrition and general counseling skills, and undernutrition management skills. Such nutrition training within the context of their practice is of paramount importance due to inadequate nutrition training in the health workers’ mainstream medical and nursing education. In-service nutrition training can take different forms such as compulsory CME, nutrition seminars, workshops, or non-compulsory continuing professional education CPD. Whatever form it takes, nutrition training has the potential to improve the quality of health workers, making them more confident and competent in this key area and thus contributing to positive changes in population nutrition.

## Authors’ Contributions

Bruno F. Sunguya conceived the research questions, designed the study, participated in the literature review and analyses, and prepared the first draft. Krishna C. Poudel refined the research question and the first draft. Linda B. Mlunde contributed to the study design, participated in the literature review, and helped to prepare the first draft. David P. Urassa revised the protocol. Junko Yasuoka participated in the preparation of the first draft and revisions. Masamine Jimba reviewed the study protocol and manuscript, and approved the submission. All authors read and approved the final version of the manuscript for submission.

## Conflict of Interest Statement

The authors declare that the research was conducted in the absence of any commercial or financial relationships that could be construed as a potential conflict of interest.
